# Endoplasmic Reticulum Stress Is Increased in Adipose Tissue of Women with Gestational Diabetes

**DOI:** 10.1371/journal.pone.0122633

**Published:** 2015-04-07

**Authors:** Stella Liong, Martha Lappas

**Affiliations:** 1 Obstetrics, Nutrition and Endocrinology Group, Department of Obstetrics and Gynaecology, University of Melbourne, Heidelberg, Victoria, Australia; 2 Mercy Perinatal Research Centre, Mercy Hospital for Women, Heidelberg, Victoria, Australia; Niigata University Graduate School of Medical and Dental Sciences, JAPAN

## Abstract

Maternal obesity and gestational diabetes mellitus (GDM) are two increasingly common and important obstetric complications that are associated with severe long-term health risks to mothers and babies. IL-1β, which is increased in obese and GDM pregnancies, plays an important role in the pathophysiology of these two pregnancy complications. In non-pregnant tissues, endoplasmic (ER) stress is increased in diabetes and can induce IL-1β via inflammasome activation. The aim of this study was to determine whether ER stress is increased in omental adipose tissue of women with GDM, and if ER stress can also upregulate inflammasome-dependent secretion of IL-1β. ER stress markers IRE1α, GRP78 and XBP-1s were significantly increased in adipose tissue of obese compared to lean pregnant women. ER stress was also increased in adipose tissue of women with GDM compared to BMI-matched normal glucose tolerant (NGT) women. Thapsigargin, an ER stress activator, induced upregulated secretion of mature IL-1α and IL-1β in human omental adipose tissue explants primed with bacterial endotoxin LPS, the viral dsRNA analogue poly(I:C) or the pro-inflammatory cytokine TNF-α. Inhibition of capase-1 with Ac-YVAD-CHO resulted in decreased IL-1α and IL-1β secretion, whereas inhibition of pannexin-1 with carbenoxolone suppressed IL-1β secretion only. Treatment with anti-diabetic drugs metformin and glibenclamide also reduced IL-1α and IL-1β secretion in infection and cytokine-primed adipose tissue. In conclusion, this study has demonstrated ER stress to activate the inflammasome in pregnant adipose tissue. Therefore, increased ER stress may contribute towards the pathophysiology of obesity in pregnancy and GDM.

## Introduction

Gestational diabetes mellitus (GDM) is characterised as any degree of glucose intolerance with first recognition during pregnancy. Risk factors for GDM include advance maternal age, racial/ethnic disparities, and obesity [1]. GDM is associated with substantial increased risks for both mother and infant [2–4]. Obesity is another significant public health concern, with a substantial increase in the rate of maternal obesity in recent times. Maternal obesity is associated with adverse health outcomes for both mother and infant during pregnancy [5]. In women, GDM and obesity-associated health risks include obesity, hypertensive disorders during pregnancy, cardiovascular diseases, recurrent GDM in future pregnancies and type 2 diabetes. In the fetus, long-term risks include obesity, diabetes, cardiovascular disease and certain cancers later in life.

Recent studies point to an important role for maternal adipose tissue in fetal growth and development. Specifically, peripheral insulin resistance, which is a normal process in human pregnancy, ensures that energy needs of the growing fetus are met [6]. However, in obese pregnant women or women with GDM however, this peripheral insulin resistance is even more pronounced [7], resulting in more substrate availability for the fetus, and thus enhanced fetal adiposity [8]. Adipose tissue also secretes a number of cytokines that may cause this peripheral insulin resistance. Notably, we have shown that the pro-inflammatory cytokine IL-1β, which is higher in adipose tissue from women with GDM [9], significantly inhibits insulin signalling in adipose tissue from pregnant women [9]. IL-1α is another important pro-inflammatory cytokine and has been linked with insulin resistance by inhibiting insulin signalling pathways [10].

*In vitro*, activation of the inflammasome is required to process pro IL-1β to an active, secreted molecule [9]. We have previously shown that women with GDM have increased activation of the inflammasome in adipose tissue [9]. Toll-like receptor (TLR) ligands and pro-inflammatory cytokines are important for the induction of IL-1β secretion in pregnant adipose tissue via inflammasome activation [9]. In contrast to IL-1β, the precise mechanism in the production of mature IL-1α remains unclear; however, there is recent evidence to suggest that IL-1α secretion may require the inflammasome [11].

Endoplasmic reticulum (ER) stress is a condition characterised by an accumulation of misfolded proteins in the ER lumen. It is triggered by various endogenous and exogenous cellular insults including environmental toxins, viral infection, and inflammation [12]. The ER stress response is initiated by the activation of three ER transmembrane proteins: PKR-like ER kinase (PERK), inositol requiring enzyme 1 (IRE1), and the activating transcription factor-6 (ATF-6). Of particular relevance is the IRE1 pathway which has been shown to induce inflammasome-dependent secretion of IL-1β [13].

ER stress is also a central feature of peripheral insulin resistance, obesity and type 2 diabetes [14,15]. There are, however, no studies on ER stress and adipose tissue in the context of GDM. Given the emerging link between ER stress-induced inflammasome activation and metabolic diseases such as obesity or type 2 diabetes, we hypothesise that ER stress is also increased in adipose tissue from obese pregnant women or women with GDM and that it mediates inflammasome activation leading to the release of IL-1 that underlies the development of insulin resistance evident in these conditions [7]. In this study we will use thapsigargin as a model of ER stress; thapsigargin activates the ER stress response by inhibiting calcium channels to deplete calcium stores in the ER, and by preventing the fusion of autophagosomes with lysosomes [16]. Studies have shown thapsigargin is also associated with increased expression of proteins involved in the ER stress response [17,18]. Therefore, the specific aims of this study are to determine (i) the effect of pre-existing maternal obesity and GDM on markers of ER stress in adipose tissue and (ii) the role of ER stress in inducing IL-1α and IL-1β secretion via the inflammasome in adipose tissue exposed to bacterial (LPS) or viral infections (poly(I:C)) and sterile inflammation (TNF-α).

## Materials and Methods

### Tissue collection and preparation

Written informed consent was obtained from all participating patients. Ethics approval was obtained from the Mercy Hospital for Women’s Research and Ethics Committee. Pregnant women were recruited to the study by a clinical research midwife. Human omental adipose tissue was obtained from women who delivered healthy, singleton infants at term (>37 weeks gestation). Indications for Caesarean section were breech presentation and/or previous Caesarean section. Women with any underlying medical conditions such as pre-existing diabetes, asthma, polycystic ovarian syndrome, preeclampsia and macrovascular complications were excluded.

Adipose tissue was obtained from the following groups: (i) normal glucose tolerant (NGT) women who entered pregnancy lean (BMI between 18-<25 kg/m^2^; n = 6 patients); (ii) NGT women who entered pregnancy obese (BMI ≥30 kg/m^2^; n = 6 patients); (iii) women with GDM were managed by diet alone (n = 6 lean and n = 6 obese); and (iv) women with GDM were managed by insulin in addition to diet (n = 6 lean and n = 6 obese). The relevant clinical details of the subjects are detailed in Table 1.

**Table 1:** Characteristics of the study group.

	Lean cohort			Obese cohort		
	NGT (*n* = 6)	GDM diet (*n* = 6)	GDM insulin (*n* = 6)	NGT (*n* = 6)	GDM diet (*n* = 6)	GDM insulin (*n* = 6)
Maternal age (years)	33.2±4.9	33.0±6.1	33.2±2.6	32.8±4.6	34.3±7.3	32.2±4.6
Pre-pregnancy maternal BMI (kg/m^2^)	22.3±1.8^†^	22.0±1.7	23.1±2.6	38.8±6.1*	36.5±5.3	41.1±9.2
Maternal BMI at delivery (kg/m^2^)	24.7±6.9^†^	26.4±3.2	27.1±3.3	41.9±5.0*	37.3±4.4*	40.1±6.0
Gestation age at birth (weeks)	38.8±0.6	38.3±0.9	38.7±0.4	38.5±0.6	38.9±0.3	38.2±0.4
Fetal birth weight (g)	3478±356	3333±423	3095±264	3678±830	3520±464	3598±327
Fetal Gender	3 Female; 3 Male	3 Female; 3 Male	4 Female; 2 Male	3 Female; 3 Male	4 Female; 2 Male	2 Female; 4 Male
Gravida	2.2±0.8	2.8±0.8	2.2±0.8	2.7±0.8	3.5±1.4	4.0±1.3
Parity	2.0±0.6	2.0±0.3	1.7±0.5	2.3±1.0	2.7±1.8	3.0±0.6
Maternal OGTT at ~28 weeks gestation						
Fasting plasma glucose (mmol/l)	4.3±0.1^†^	5.4±0.3*	5.2±1.8*	4.8±0.3*	5.2±0.8	6.3±0.9^†^ ^§^
1 h plasma glucose (mmol/l)	7.4±1.6	10.8±1.3*	11.1±1.0*	7.9±1.3	10.9±0.8^†^	11.3±1.1^†^
2 h plasma glucose (mmol/l)	6.6±0.7	9.0±1.9*	9.8±1.0*	5.9±0.8	10.2±0.7^†^	10.5±1.5^†^

Values represent mean ± SEM (Student’s t-test)

BMI, body mass index

*P<0.05 vs. NGT lean

^†^ *P*<0.05 vs. NGT obese

^§^ *P*<0.05 vs. obese NGT diet

Women with GDM were diagnosed according to the criteria of the Australasian Diabetes in Pregnancy Society (ADIPS) by either a fasting venous plasma glucose concentrations of ≥5.5 mmol/l glucose, and/or ≥8.0 mmol/l glucose 2 h after a 75 g oral glucose load at approximately 28 weeks gestation. Women were controlled by diet if their fasting glucose readings were maintained below 5.5 mmol/l over a 2 week period post diagnosis. Women with fasting glucose readings greater than 5.5 mmol/l were placed on insulin for optimal glucose control. All pregnant women were screened for GDM, and women participating in the normal group had a negative screen. Adipose tissue was processed within 15 min of collection, thoroughly washed in ice-cold PBS to remove any blood. Dissected fragments were stored at -80°C until assayed as detailed below.

### Tissue explant culture

Tissue explants were performed to determine the effect of ER stress induction (using thapsigargin) on inflammasome activation in pregnant adipose tissue. For these studies, adipose tissue was obtained from NGT non-obese pregnant women, and tissue explants were performed as previously described [9]. Briefly, adipose tissues was finely diced and placed in DMEM at 37°C in a humidified atmosphere of 21% O_2_ and 5% CO_2_ for 1 h. Tissues were blotted dry on sterile filter paper and transferred to 24-well tissue culture plates (100 mg wet weight/well). The explants were incubated in 1 ml DMEM containing 100 U/ml penicillin G and 100 μg/ml streptomycin. Tissues were incubated in the absence or presence of 10 μg/ml LPS (derived from *E*.*coli* strain 026:B6 (Sigma-Aldrich; St. Louis, MO)), 20 μg/ml poly(I:C) or 1 ng/ml TNF-α for 18 h, followed by incubation with 30 μM thapsigargin for 2 h. Concentrations of LPS and TNF-α were based on our previous studies [9]. Additional experiments were also performed whereby adipose tissue was pre-treated with 20 μM of the caspase-1 inhibitor Ac-YVAD-CHO, 10 μM of the connexin/pannexin-1 channel blocker carbenoxolone, or with two anti-diabetic drugs, 25 μM glibenclamide or 0.5 mM metformin prior to the addition of LPS, poly(I:C) or TNF-α. The concentration of thapsigargin and inhibitors was based on an initial dose-response and past studies [9]. After the final incubation, medium was collected and assessment of cytokine concentrations was performed by ELISA, whereas qRT-PCR was used to assess cytokine gene expression in adipose tissue. Each treatment was performed from adipose tissue obtained from at least five patients.

### RNA extraction and quantitative RT-PCR (qRT-PCR)

RNA extraction and qRT-PCR was performed as previously described [9]. Briefly, total RNA was extracted from tissues using TRIsure according to manufacturer’s instructions (Bioline; Alexandria, NSW, Australia). RNA concentration and purity were measured using a NanoDrop ND1000 spectrophotometer (Thermo Fisher Scientific; Scoresby, Vic, Australia). RNA was converted to cDNA the Tetro cDNA synthesis kit (Bioline) according to the manufacturer’s instructions. The cDNA was diluted fifty-fold, and 4 μl of this was used to perform RT-PCR using SensiFAST SYBR (Bioline) and 100 nM of pre-designed and validated QuantiTect primers (Qiagen, Chadstone Centre, Vic, Australia). The RT-PCR was performed using a CFX384 Real-Time PCR detection system from Bio-Rad Laboratories (Bio-Rad Laboratories; Gladesville, NSW, Australia). Average gene Ct values were normalised to the average 18S Ct values of the same cDNA sample. Fold differences were determined using the comparative Ct method. As previously reported [19,20], for the explant studies, there was a large variability in the response to LPS, poly(I:C) or TNF-α which is normal for tissues derived from different patients. Thus, fold change was calculated relative to LPS, poly(I:C) or TNF-α, which was set at 1.

### Western blotting

Tissue lysates and Western blotting were prepared as previously described [9]. Forty micrograms of protein was separated on 7.5% polyacrylamide gels (Bio-Rad Laboratories) and transferred onto nitrocellulose. Blots were cut into three sections of MW ranges: 250–100 kD, 100–65 kD and 30–65 kD. The 250–100 kD section was probed with rabbit polyclonal anti-IRE1α (1:1000 dilution; Cell Signalling Technology, Danvers, MA, USA); the 100–65 kD section was probed with rabbit polyclonal anti-GRP78 (1:1000 dilution; GeneTex, Irvine, CA) and the 20–65 kD section was probed with mouse monoclonal anti-XBP-1s (1 μg/ml; BioLegend, San Diego, CA) antibodies. Protein expression was identified by comparison with the mobility of protein standard. Membranes were viewed and analysed using the ChemiDoc XRS system (Bio-Rad Laboratories). Semi-quantitative analysis of the relative density of the bands in Western blots was performed using the Image Lab analysis software (Version 3, Bio-Rad Laboratories). Ponceau S staining was used as a loading control. In the obese study, the data was normalised to the NGT lean group. In the GDM study, the data was normalised to the BMI-matched NGT group.

### Cytokine immunoassays

Assessment of IL-6 release was performed using CytoSet sandwich ELISA according to the manufacturer’s instructions (Life Technologies; Mulgrave, Vic, Australia). The limit of detection of the IL-6 assay was 16 pg/ml. The concentration of IL-1α and IL-1β in the media was performed by sandwich ELISA according to the manufacturer’s instructions (R&D Systems, Minneapolis, MN). The limit of detection of the IL-1α and IL-1β assays was 7.8 and 2 pg/ml, respectively. All data were corrected for total protein and expressed as either pg or ng per mg protein. The protein content of tissue homogenates was determined using BCA protein assay (Pierce, Rockford, IL), using BSA as a reference standard, as previously described [21]. The calculated inter-assay and intra-assay coefficients of variation (CV) were all less than 10%.

### Statistical analysis

**Fig 1:**
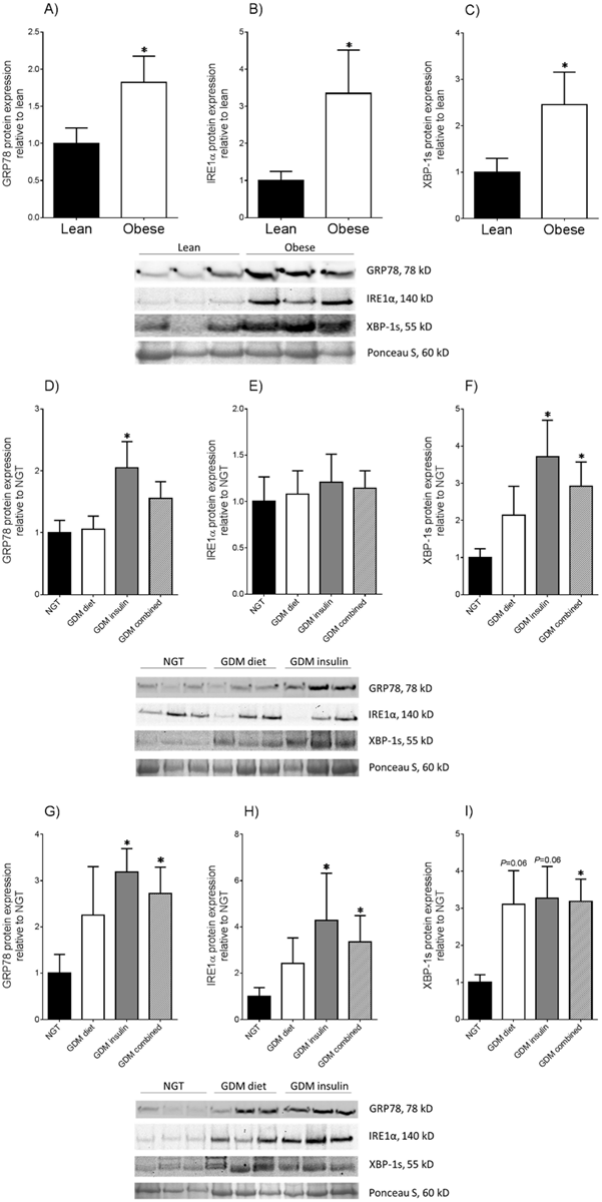
ER stress is increased in adipose tissue from obese pregnant women and with GDM. Adipose tissue was obtained from NGT lean (n = 6 patients) and obese (n = 6 patients) pregnant women at the time of term Caesarean section. The protein abundance of **(A)** GRP78, **(B)** IRE1α and **(C)** XBP-1s was analysed by Western blot. Protein abundance was normalised to Ponceau S staining and the fold change was calculated relative to the lean group. Data is presented as mean ± SEM. *P<0.05 vs. lean (Student’s t-test). Representative Western blot from six patients (3 obese and 3 lean) is also shown. Adipose tissue were also obtained from **(D-F)** lean and **(G-I)** obese pregnant women with NGT (n = 6 patients per group), diet-controlled GDM (n = 6 patients per group), insulin-controlled GDM (n = 6 patients) and combined GDM (n = 12 patients per group) at the time of term Caesarean section. The protein abundance of **(D-G)** GRP78, **(E-H)** IRE1α and **(F-I)** XBP-1s was analysed by Western blot. Protein abundance was normalised to Ponceau S staining and the fold change was calculated relative to the NGT group. Data is presented as mean ± SEM. *P<0.05 vs. NGT (Student’s t-test). Representative Western blot from nine patients (3 NGT, 3 GDM diet and 3 GDM insulin) is also shown.

**Fig 2:**
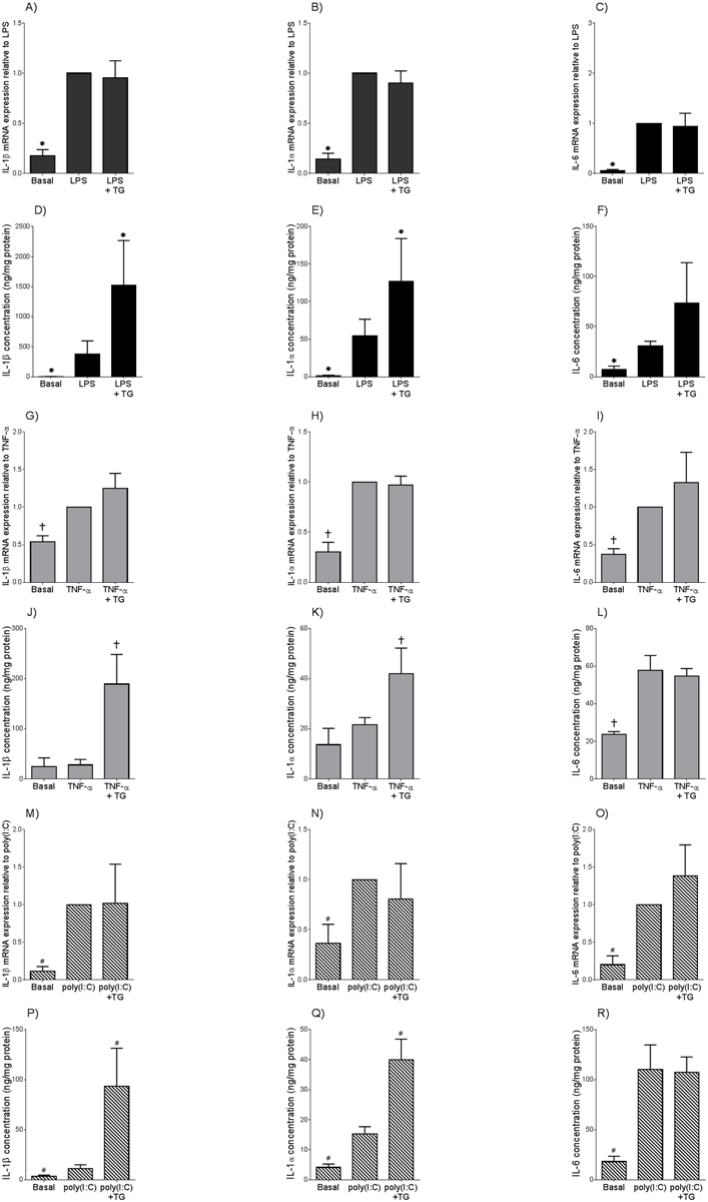
Thapsigargin induces enhanced IL-1α and IL-1β secretion but not gene expression in adipose tissue primed with LPS, poly(I:C) or TNF-α. Adipose tissue was incubated in the absence or presence of **(A-F)** 10 μg/ml LPS (n = 6 patients), **(G-L)** 20 μg/ml poly(I:C) (n = 6 patients), or **(M-R)** 10 ng/ml TNF-α. After 18 h incubation, tissues were incubated with 30 μM thapsigargin (TG) for a further 2 h. IL-1β, IL-1α, and IL-6 mRNA expression was analysed by qRT-PCR and normalised to 18S and fold change expressed relative to basal levels. The incubation medium was also assayed forIL-1β, IL-1α and IL-6 concentration by ELISA. Each bar represents mean ± SEM. *P<0.05 vs. LPS (one way ANOVA). #*P*<0.05 vs. poly(I:C) (one-way ANOVA). †*P*<0.05 vs. TNF-α (one-way ANOVA).

**Fig 3:**
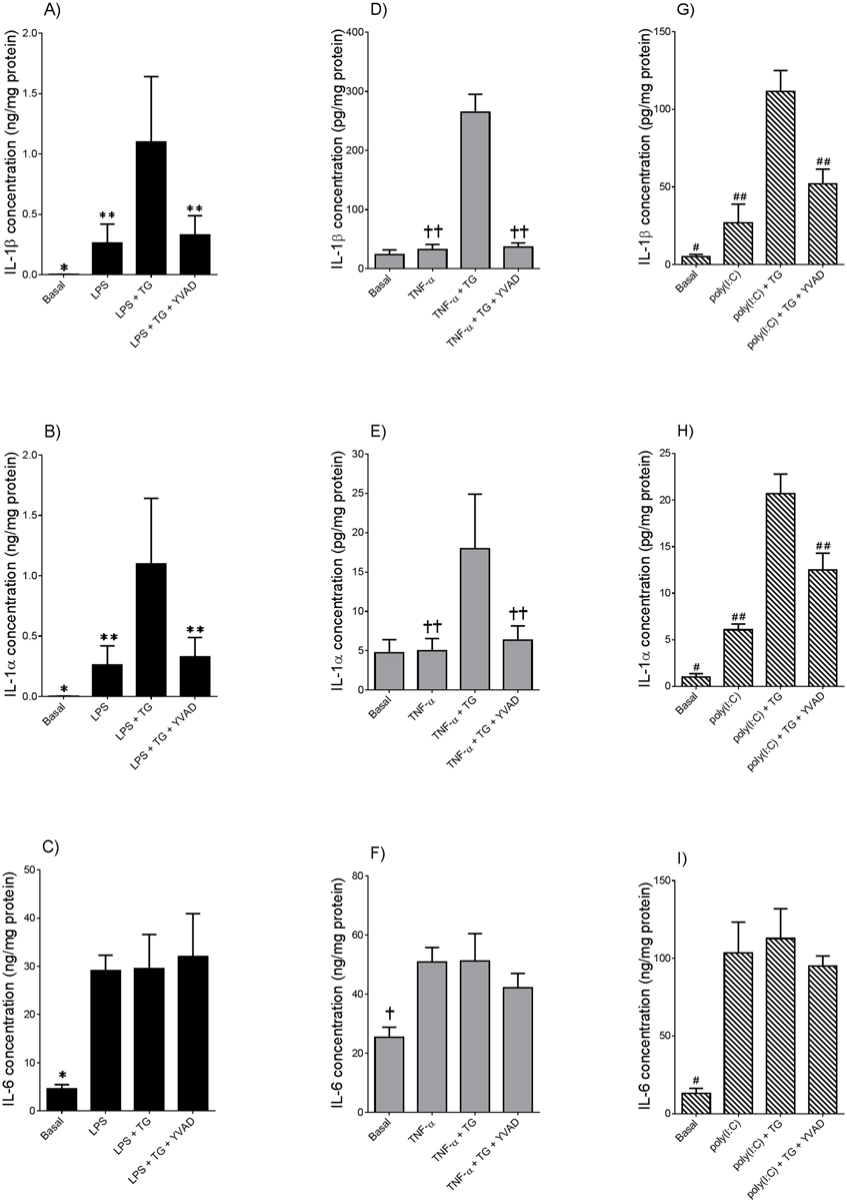
Caspase-1 regulates thapsigargin-induced IL-1α and IL-1β secretion in tissues primed with LPS, poly(I:C) or TNF-α. Adipose tissue was incubated in the absence or presence of 10 μM Ac-YVAD-CHO (YVAD) for 60 min prior to the addition of **(A-C)** 10 μg/ml LPS, **(D-F)** 20 μg/ml poly(I:C) or **(G-I)** 10 ng/ml TNF-α. After 18 h incubation, tissues were incubated with 30 μM thapsigargin (TG) for a further 2 h (n = 6 patients per treatment). The incubation medium was assayed for IL-1α, IL-1β and IL-6 concentration by ELISA. Each bar represents mean concentration ± SEM. *P<0.05 vs. LPS (one way ANOVA); **P<0.05 vs. LPS + TG (one way ANOVA); #*P*<0.05 vs. poly(I:C) (one-way ANOVA); ##*P*<0.05 vs. poly(I:C) + TG (one-way ANOVA); †*P*<0.05 vs. TNF-α (one-way ANOVA); ††*P*<0.05 vs. TNF-α + TG (one-way ANOVA).

**Fig 4:**
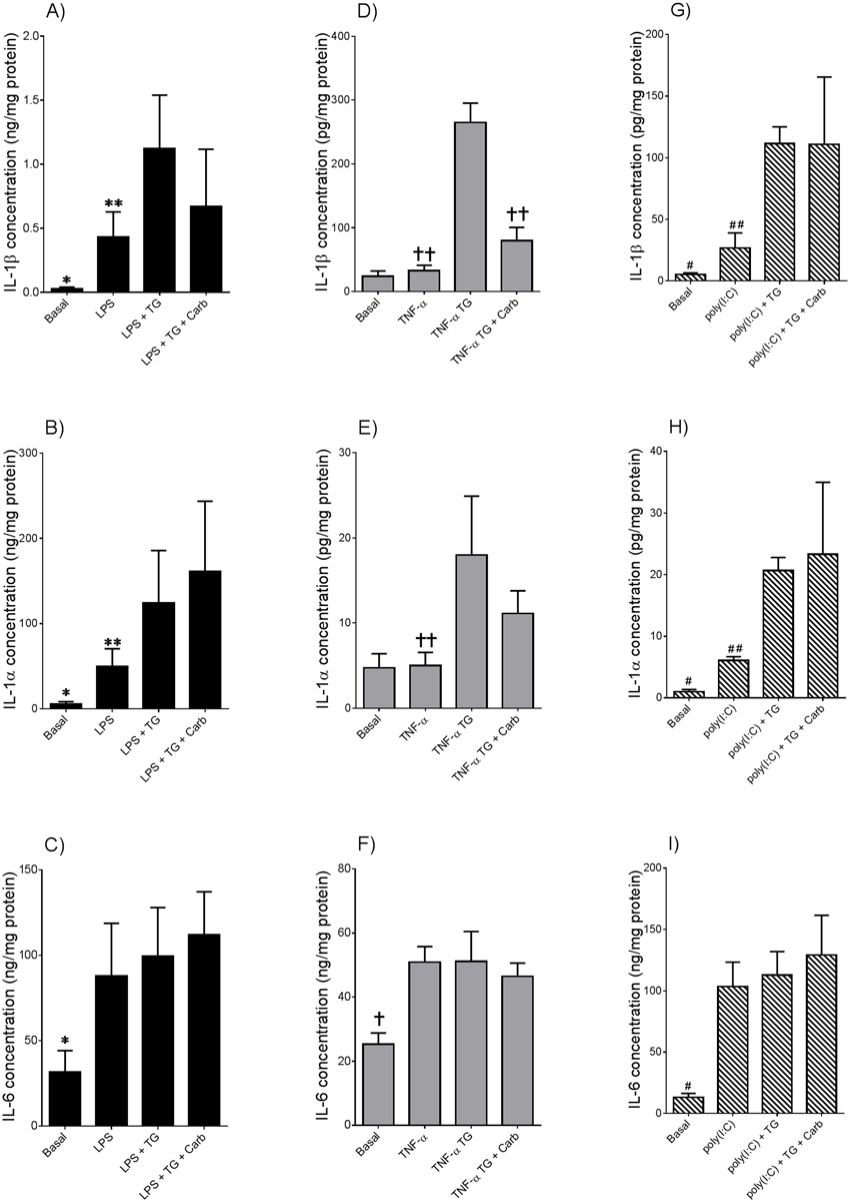
Pannexin-1 regulates thapsigargin-induced IL-1β but not IL-1α secretion in tissues primed with LPS, poly(I:C) or TNF-α. Adipose tissue was incubated in the absence or presence of 10 μM carbenoxolone (Carb) for 60 min prior to the addition of **(A-C)** 10 μg/ml LPS, **(D-F)** 20 μg/ml poly(I:C) or **(G-I)** 10 ng/ml TNF-α. After 18 h incubation, tissues were incubated with 30 μM thapsigargin (TG) for a further 2 h (n = 6 patients per treatment). The incubation medium was assayed for IL-1α, IL-1β and IL-6 concentration by ELISA. Each bar represents mean concentration ± SEM. *P<0.05 vs. LPS (one way ANOVA); **P<0.05 vs. LPS + TG (one way ANOVA); #*P*<0.05 vs. poly(I:C) (one-way ANOVA); ##*P*<0.05 vs. poly(I:C) + TG (one-way ANOVA); †*P*<0.05 vs. TNF-α (one-way ANOVA); ††*P*<0.05 vs. TNF-α + TG (one-way ANOVA).

**Fig 5:**
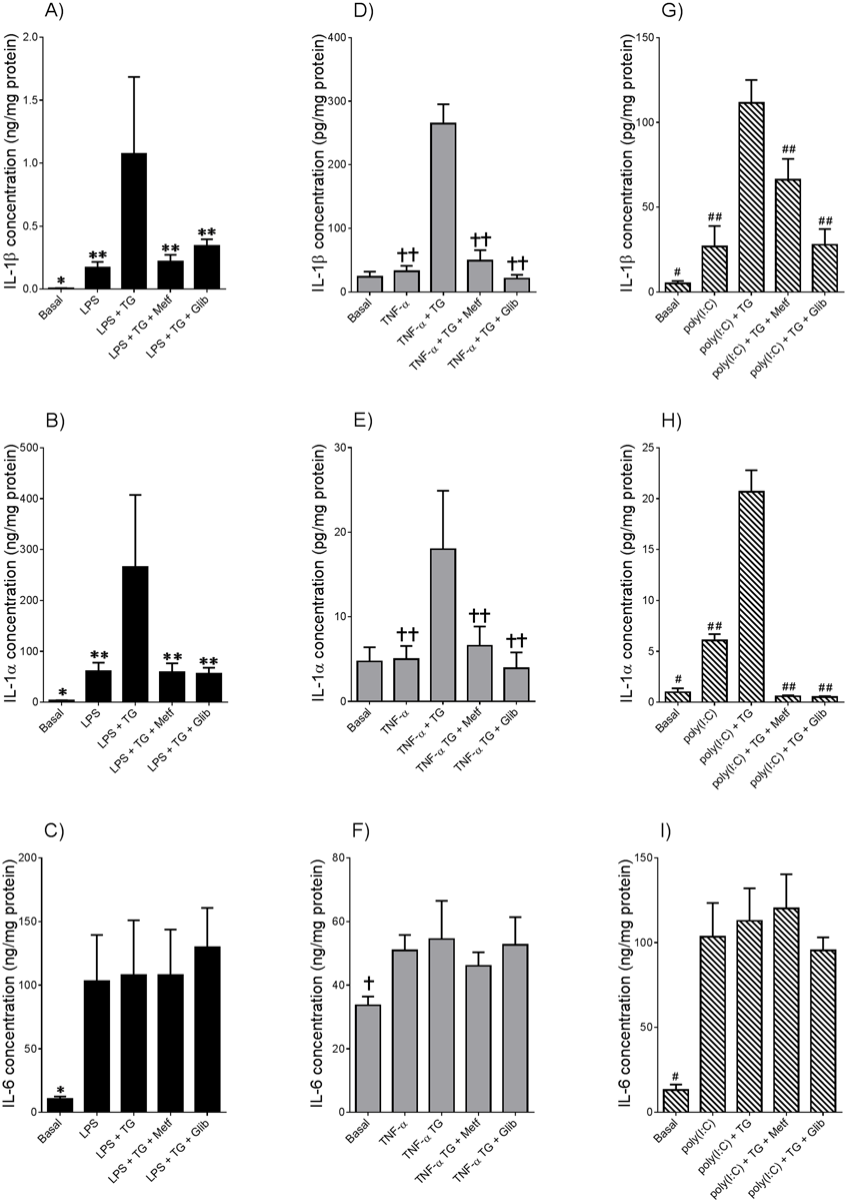
Anti-diabetic drugs inhibits thapsigargin-induced IL-1α and IL-1β secretion in tissues primed with LPS, poly(I:C) or TNF-α. Adipose tissue was incubated in the absence or presence of 0.5 mM metformin (Metf) or 25 μM glibenclamide (Glib)for 60 min prior to the addition of **(A-C)** 10 μg/ml LPS, **(D-F)** 20 μg/ml poly(I:C) or **(G-I)** 10 ng/ml TNF-α. After 18 h incubation, tissues were incubated with 30 μM thapsigargin (TG) for a further 2 h (n = 6 patients per treatment). The incubation medium was assayed for IL-1α, IL-1β and IL-6 concentration by ELISA. Each bar represents mean concentration ± SEM. *P<0.05 vs. LPS (one way ANOVA); **P<0.05 vs. LPS + TG (one way ANOVA); #*P*<0.05 vs. poly(I:C) (one-way ANOVA); ##*P*<0.05 vs. poly(I:C) + TG (one-way ANOVA); †*P*<0.05 vs. TNF-α (one-way ANOVA); ††*P*<0.05 vs. TNF-α + TG (one-way ANOVA).

Statistics was performed on the normalised data unless otherwise specified. All statistical analyses were undertaken using GraphPad Prism Version 6 (GraphPad Software, La Jolla, CA). For Fig 1, an unpaired Student’s t-test was used to assess statistical significance between normally distributed data; otherwise, the nonparametric Mann-Whitney U test was used. For Figs 2–5, the homogeneity of data was assessed by the Bartlett test, and when significant, the data were logarithmically transformed before further analysis using a one-way ANOVA (using LSD correction to discriminate among the means). Statistical significance was ascribed to *P* value <0.05. Data were expressed as mean ± standard error of the mean (SEM).

## Results

### ER stress is increased in adipose tissue from obese women

Adipose tissue was obtained from 12 NGT women at the time of term Caesarean section (in the absence of labour). Participants were further classified as being either lean (n = 6 patients) or obese (n = 6 patients) based on pre-pregnancy BMI. Demographic data of all participants involved in this study are summarised in Table 1. There were no significant differences in maternal age, gestational age at delivery, and fetal birth weight between the two groups. As expected, maternal BMI (both pre-pregnancy and at delivery) was significantly higher in the obese patients compared to the lean patients. There was no difference in the fasting OGTT between NGT lean and obese patients.

GRP78, XBP-1s and IRE1α are commonly used indicators of ER stress [22,23]. As presented in Fig 1, the protein expression of these ER stress markers was assessed by Western blotting in adipose tissue of NGT pregnant women. Compared to lean patients, GRP78 (Fig 1A), IRE1α (Fig 1B) and XBP-1s (Fig 1C) protein expression were significantly elevated in adipose tissue of obese NGT patients.

### ER stress is increased in adipose tissue from women with GDM

To determine the effect of GDM on ER stress protein expression, adipose tissue was obtained from NGT pregnant women (n = 12 patients; 6 lean and 6 obese) and BMI-matched women with diet-controlled GDM (n = 12 patients; 6 lean and 6 obese) or with insulin-controlled GDM (n = 12 patients; 6 lean and 6 obese). Demographic data of the participants involved in this investigation are summarised in Table 1. There were no significant differences in maternal age, maternal BMI, gestational age at delivery, and fetal birth weight between the GDM and NGT groups. In the lean cohort, fasting, one-hour and two-hour glucose concentrations during the pregnancy OGTT was significantly higher in GDM patients (both diet and insulin) compared to NGT patients. The fasting, one-hour and two-hour OGTT readings in obese GDM insulin patients was significantly higher compared to obese NGT patients. No significant difference was observed in the fasting OGTT reading between obese GDM diet and obese NGT patients, whereas the one-hour and two-hour OGTT readings were significantly higher in obese GDM diet patients compared to obese NGT patients.

In the lean cohort, GRP78 protein expression was significantly higher in GDM insulin compared to NGT patients, whereas no change in GRP78 expression was detected between GDM diet and NGT patients (Fig 1D). As shown in Fig 1F, a non-significant increase in XBP-1s expression was detected in GDM diet patients when compared to NGT patients (*P* = 0.09). On the other hand, adipose tissue expression of XBP-1s was significantly greater in both GDM insulin and GDM combined groups when compared to NGT patients.

In the obese cohort, patients in the GDM insulin and GDM combined groups had significantly elevated protein expression levels of GRP78 and IRE1α when compared to NGT patients (Fig 1G and 1H). In contrast, there was no significant difference in GRP78 and IRE1α protein expression levels between GDM diet and NGT patients (Fig 1G and 1H). As shown in Fig 1I, there was a non-significant increase in the expression of XBP-1s in adipose tissue obtained from GDM diet and GDM insulin patients when compared to NGT patients (*P* = 0.06). However, when the diet and GDM groups were combined, XBP-1s was significantly higher when compared to adipose tissues obtained from NGT patients.

Notably, for both the lean and obese cohort, there was no difference in GRP78, IRE1α and XBP-1s protein expression between GDM women managed by diet or insulin.

### The ER stress activator thapsigargin induces IL-1α and IL-1β secretion from adipose tissue primed with LPS, poly(I:C) or TNF-α

We have previously shown that two signals are required for the upregulated secretion of IL-1β in pregnant adipose tissue; the first signal, such as the TLR4 ligand LPS or the pro-inflammatory cytokine TNF-α, induces the transcription and translation of pro IL-1β via NF-κB [9]. A second signal is then required to activate caspase-1 in order to process pro IL-1β into the mature and secreted form of IL-1β. Thus, we sought to determine if activation of ER stress using thapsigargin induces inflammasome activation in adipose tissues from primed with TNF-α, or with TLR ligands LPS (TLR4 agonist) and poly(I:C) (TLR3 agonist). It should be noted that experiments all subsequent experiments were performed on adipose tissue obtained from NGT non-obese pregnant women.

LPS induced a significant increase in both IL-1α and IL-1β mRNA expression (Fig 2A and 2B) and secretion (Fig 2D and 2E) in adipose tissue. The addition of thapsigargin resulted in a further increase in IL-1α and IL-1β release whilst having no effect on gene transcription. The specificity of thapsigargin to upregulate IL-1 secretion was assessed by analysing IL-6, given that IL-6 secretion is not dependent on the inflammasome activity. As expected, LPS induced a significant increase in IL-6 mRNA expression (Fig 2C) and release (Fig 2F). There was, however, no effect of thapsigargin on IL-6 mRNA expression (Fig 2C) and secretion (Fig 2F) in the presence of LPS. Of note, thapsigargin had no effect on the gene expression or secretion of IL-1α, IL-1β and IL-6 in unprimed adipose tissue (data not shown).

Poly(I:C) treatment of adipose tissue also induced a significant increase in IL-1α, IL-1β and IL-6 mRNA expression (Fig 2G–2I) and secretion (Fig 2J–2L). Poly(I:C)-induced secretion of IL-1α and IL-1β, but not IL-6, was significantly augmented following thapsigargin treatment (Fig 2I–2L). On the other hand, thapsigargin had no effect on poly(I:C)-stimulated IL-1α, IL-1β and IL-6 mRNA expression (Fig 2G–2I).

In contrast to LPS and poly(I:C) treatments, TNF-α increased IL-1α and IL-1β mRNA expression (Fig 2M and 2N) but not secretion (Fig 2P and 2Q) in pregnant adipose tissue. Thapsigargin did not have an additive effect on IL-1α and IL-1β mRNA expression in TNF-α-stimulated adipose tissues (Fig 2M and 2N); however, secretion of both IL-1α and IL-1β was significantly increased following thapsigargin treatment (Fig 2P and 2Q). TNF-α also increased both IL-6 mRNA expression (Fig 2O) and secretion (Fig 2R) in pregnant adipose tissue, however, there was no additional effect following treatment with thapsigargin.

### Caspase-1 regulates thapsigargin induced IL-1α and IL-1β secretion from adipose tissue primed with LPS, poly(I:C) or TNF-α

In our previous studies, we have shown that suppression of caspase-1 activity ameliorated IL-1β secretion in pregnant adipose tissue primed with LPS or TNF-α [9]. Thus, the next aim was of this study was to determine if caspase-1 is involved in the genesis of thapsigargin-induced IL-1α and IL-1β secretion in LPS, TNF-α or poly(I:C) primed adipose tissue.

As expected, thapsigargin induced a significant increase in IL-1α and IL-1β, but not IL-6 secretion, in adipose tissue primed with LPS (Fig 3A–3C), poly(I:C) (Fig 3D–3F) or TNF-α (Fig 3G–3I). Pre-treatment with the specific caspase-1 inhibitor Ac-YVAD-CHO significantly attenuated thapsigargin-induced IL-1α and IL-1β in adipose tissue primed with LPS (Fig 3A and 3B), poly(I:C) (Fig 3D and 3E) or TNF-α (Fig 3G and 3H). The specificity of Ac-YVAD-CHO to suppress caspase-1-dependent secretion of IL-1α and IL-1β was confirmed by having no effect on IL-6 secretion (Fig 3C,3F and 3I).

### Thapsigargin induces IL-1β but not IL-1α secretion via pannexin-1 channel from adipose tissue primed with LPS, poly(I:C) and TNF-α

We have previously shown the pannexin-1 channel to act as another second signal activator of the inflammasome in adipose tissue [9]. Therefore, the next aim of this study was to determine the effect of the pannexin-1 inhibitor carbenoxolone on IL-1α and IL-1β secretion from adipose tissue treated with LPS, poly(I:C) or TNF-α.

Pre-treatment with carbenoxolone significantly attenuated thapsigargin-induced IL-1β but not IL-1α secretion in adipose tissue primed with LPS (Fig 4A and 4B), poly(I:C) (Fig 4D and 4E) or TNF-α (Fig 4G and 4H). The specificity of carbenoxolone to inhibit IL-1β secretion was confirmed by having no effect on IL-6 secretion (Fig 4C,4F and 4I).

### Anti-diabetic drugs metformin and glibenclamide attenuates thapsigargin induced IL-1α and IL-1β secretion from adipose tissue primed with LPS, poly(I:C) or TNF-α

Studies have identified the anti-diabetic drugs metformin and glibenclamide as modulators of inflammasome-dependent secretion of IL-1β in macrophages [24–26]. Following on from these findings, the final aim of this study was to assess the effect of metformin and glibenclamide on adipose secretion of IL-1α and IL-1β following exposure to LPS, TNF-α or poly(I:C).

Pre-treatment with metformin or glibenclamide significantly attenuated thapsigargin-enhanced IL-1α and IL-1β secretion in adipose tissue primed with LPS (Fig 5A and 5B), poly(I:C) (Fig 5D and 5E) or TNF-α (Fig 5G and 5H). Neither metformin nor glibenclamide had any effect on IL-6 secretion, confirming their specificity for inflammasome activation (Fig 5C,5F and 5I).

## Discussion

This study, for the first time, demonstrates the IRE1 arm of the ER stress pathway is increased in adipose tissue from obese pregnant women and women with GDM, as evidenced by the increased expression of ER stress proteins GRP78, IRE1α and/or XBP-1s. We also report that activation of ER stress is associated with increased activation of the inflammasome. That is, activation of ER stress by thapsigargin in pregnant adipose tissues primed with LPS, poly(I:C) or TNF-α, induced IL-1β and IL-1α secretion but not transcription; an effect which was suppressed using inhibitors of pannexin-1 and caspase-1. Furthermore, anti-diabetic drugs metformin and glibenclamide also suppressed thapsigargin-induced inflammasome activation in tissues primed with LPS, poly(I:C) or TNF-α. Notably, there was no effect by ER stress activation or inflammasome inhibitors on IL-6 secretion. Given the central role of IL-1 in mediating inflammation, insulin resistance and obesity [27], these findings suggest ER stress may contribute to the pathophysiology of GDM and obesity in pregnancy via aberrant inflammasome activation.

A number of metabolic diseases such as obesity and diabetes are associated with increased ER stress [14,15]. Markers of ER stress including GRP78 are upregulated in adipose tissue of obese mice [28]. Mice fed with a high-fat diet supplemented with ER stress inhibitor 4-phenylbutyric acid (4-PBA), demonstrated a significant reduction in weight gain [29]. Moreover, treatment of obese and diabetic mice with ER stress inhibitors improved insulin sensitivity in the liver, muscles and adipose tissues [15]. In this study we have also described, for the first time, ER stress proteins IRE1α, GRP78 and XBP-1s were significantly increased in pregnant adipose tissue of obese NGT women compared to BMI-matched lean NGT women. ER stress proteins were also increased in women with GDM compared to NGT women. Furthermore, comparisons with BMI-matched NGT women found ER stress proteins were consistently higher in obese GDM women than in lean GDM women. Most notably was the significant increase in GRP78 expression in adipose tissues of patients with insulin-controlled GDM irrespective of BMI. The severity in the metabolic disturbance in GDM women who require insulin treatment as opposed to diet-controlled GDM women may explain the observed difference in the expression of ER stress proteins between the two GDM groups when compared to BMI-matched NGT women. Taken together, our findings suggest that ER stress may have a role in the pathophysiology of obesity in pregnancy and GDM.

Maternal obesity and GDM are associated with inflammasome activation and/or increased IL-1β [9]. IL-1β induces peripheral insulin resistance [9], which contributes to the pathophysiology of GDM or pre-existing maternal obesity [7]. High levels of IL-1β are also associated with endothelial cell dysfunction [30]. Notably, we have previously described endothelial cell function is impaired in adipose tissue from women with pre-existing obesity and GDM [31]. Although the role of IL-1α in obese and GDM pregnancies has not been well characterised, there is growing evidence to implicate IL-1α to have a role in diabetes and obesity [32–34].

Emerging studies have shown ER stress is involved in inflammasome activation and subsequent IL-1 secretion in a number of different cell types including adipocytes [16,35]. *In vivo*, mice injected with the ER stress inducer tunicamycin have shown significant increases in serum IL-1β [36] while inhibition of ER stress in diabetic mice attenuated ER stress induced-inflammasome activation [16]. Similarly, *in vitro* studies have reported ER stress suppression is associated with a reduction in IL-1β secretion [36]. Thus, we sought to determine if increased ER stress, which is evident in adipose tissue of obese pregnant women and women with GDM, may contribute to IL-1 production via the inflammasome. It is well characterised that two signals are required for optimal IL-1β secretion. First, a pro-inflammatory signal is required for IL-1β gene transcription via NF-κB. Second, inflammasome-mediated capase-1 activation is then required for the subsequent processing of pro IL-1β to the mature and secreted form of IL-1β. Metabolic endotoxemia, viral infections and inflammation are all described to initiate the pathophysiology involved in obesity and insulin resistance [37–39]. Therefore, to assess the role of ER stress on inflammasome activation in this study, LPS, poly(I:C) and TNF-α was used as the first signal activators and thapsigargin as the second signal activator for inflammasome-mediated IL-1 production. LPS, poly(I:C) and TNF-α all induced IL-1α and IL-1β gene transcription with minimal IL-1α and IL-1β cytokine release. However co-stimulation with thapsigargin induced a further increase in IL-1α and IL-1β secretion with no effect on gene transcription. To confirm that thapsigargin was specifically activating the inflammasome, IL-6 secretion was also measured. Unlike IL-1α and IL-1β, IL-6 secretion is not dependent on the inflammasome and in keeping with this our data showed thapsigargin had no additive effect on IL-6 secretion in the presence of LPS, TNF-α or poly(I:C). It is of note that treatment with thapsigargin alone did not induce IL-1α or IL-1β secretion on unstimulated adipose tissue. Together, these findings suggest that ER stress activation of the inflammasome requires pre-existing inflammation.

In order to examine in more detail the effects of thapsigargin-induced ER stress on the inflammasome, two specific inhibitors were used to target different components of the inflammasome pathway, namely caspase-1 and the upstream cell-surface receptor pannexin-1. Activation of caspase-1 is required for optimal IL-1α and IL-1β maturation and secretion [9,40], with growing evidence implicating uncontrolled caspase-1 activation to be responsible for the pathophysiology of obesity and type 2 diabetes [9]. The formation of the inflammasome complex induces caspase-1 activation; post-translational modification of the constitutively expressed pro-enzyme, procaspase, generates the subunits p10 and p20 to form the active caspase-1 enzyme. Indeed, caspase-1 activation is increased in adipose tissue of GDM women [9]. Further, the caspase-1 peptide inhibitor AC-YVAD-CHO significantly decreased LPS-induced IL-1β release, but not IL-6 or IL-8, in pregnant adipose tissue primed with LPS [9]. Given that IL-1α and IL-1β secretion is dependent on caspase-1 [9,11], the ability of caspase-1 to induce IL-1 secretion during ER stress was of interest to this study. We have shown in this study that inhibition of caspase-1 significantly attenuated thapsigargin-induced IL-1α and IL-1β secretion in LPS, TNF-α or poly(I:C) primed pregnant adipose tissue. In contrast to IL-1 production, IL-6 was not affected by caspase-1 inhibition. Collectively, these findings demonstrate that caspase-1 is required for optimal IL-1 secretion but not for the secretion of other cytokines.

Assembly of the inflammasome complex can be initiated via activation of two transmembrane bound receptors P2X7 and pannexin-1. Once activated, a complex is formed between these two receptors, creating a large pore channel at the cell surface [41]. This pore structure allows for the cytoplasmic entry of ligands for inflammasome activation [42]. Given that pannexin-1 is involved in mediating the release of mature IL-1β [43], the ability of pannexin-1 to mediate IL-1 production during ER stress was also of interest to this study. This study demonstrates the use of the pannexin-1 antagonist carbenoxolone to significantly attenuate thapsigargin-induced IL-1β secretion in adipose tissue primed with LPS, TNF-α or poly(I:C). Thapsigargin-induced IL-1α release, however, was not dependent on pannexin-1 activity. Taken together, our findings indicate a differential requirement for the inflammasome-dependent secretion of IL-1α in comparison to IL-1β.

Collectively, our results demonstrate IL-1α and IL-1β secretion in adipose tissue is tightly controlled by two signals. The first signal required is mediated by an exogenous stimuli, such as the TLR ligands LPS (TLR4) and poly(I:C) (TLR3) and the pro-inflammatory cytokine TNF-α, triggering the gene transcription of IL-1 and a modest production of mature IL-1. A second signal caused by ER stress results in the activation of pannexin-1 leading to caspase-1 activation and subsequent increase in production and secretion of mature IL-1α and IL-1β.

In addition to well-known inhibitors of inflammasome activity, this study has also investigated the effects of two anti-diabetic drugs metformin and glibenclamide on their role in regulating ER stress-induced inflammasome activation. Metformin and glibenclamide are two oral anti-diabetic drugs commonly used for the treatment of type 2 diabetes by improving insulin responsiveness [44,45]. Studies have shown metformin and glibenclamide to inhibit inflammasome activation [46]. Similarly in this study, metformin and glibenclamide significantly attenuated inflammasome activation in adipose tissue primed with LPS, poly(I:C) and TNF-α.

Given that prolonged ER stress is involved in the progression of many metabolic diseases including diabetes, the ability of metformin and glibenclamide to inhibit ER stress-mediated inflammasome activation suggests a therapeutic role for these drugs in the treatment of GDM. In the past, the use of metformin as a treatment for women with GDM has been restricted amidst concerns on the potential risk of adverse maternal and fetal side-effects as it is known to cross the placenta [47]. Recent and quite promising data, however, have demonstrated that metformin is particularly advantageous compared to insulin therapy in the treatment of GDM in overweight or obese women with respect to maternal weight gain and neonatal outcomes [48]. In addition, the use of metformin in pregnancy has shown no risk to neonates and children up to 2 years of age [49,50]. In contrast to metformin, glibenclamide does not cross the placenta [51]. A meta-analysis study on the use of glibenclamide in GDM women found the drug to be as effective as insulin therapy however the risk of developing neonatal hypoglycemia, high fetal birth weight, and macrosomia were increased in women treated with glibenclamide [52].

Although we have shown that metformin and glibenclamide are able to suppress ER stress-induced inflammasome activation in primed adipose tissue, a limitation of this study is that it remains uncertain whether metformin and glibenclamide inhibits the inflammasome by directly acting on the signalling pathways involved in its activation or have more upstream roles by attenuating ER stress.

In summary, ER stress plays a crucial role in inflammasome activation in adipose tissue of pregnant women. The findings presented in this study shows ER stress is increased in adipose tissue of obese pregnant women and in pregnancies complicated by GDM. We found that activation of ER stress could induce IL-1α and IL-1β secretion in pregnant adipose tissue primed with LPS, poly(I:C) or TNF-α. IL-1β is a major contributor to the pathophysiology of obesity in pregnancy and GDM [7,9]. Therefore inhibiting ER stress induced IL-1 production may be a potential therapeutic in improving pregnancy complications associated with maternal obesity and GDM. Indeed, the administration of anti-diabetic drugs metformin and glibenclamide were found to successfully supress inflammasome activation induced by ER stress. Given that metformin drugs is widely used for the treatment of type 2 diabetes and has recently been found to have no adverse effect on neonates, its prophylactic application for the prevention of GDM represents an attractive avenue for further research.
